# Isolated Assessment of Translation or Rotation Severely Underestimates the Effects of Subject Motion in fMRI Data

**DOI:** 10.1371/journal.pone.0106498

**Published:** 2014-10-21

**Authors:** Marko Wilke

**Affiliations:** 1 Department of Pediatric Neurology and Developmental Medicine, Children's Hospital, University of Tübingen, Tübingen, Germany; 2 Experimental Pediatric Neuroimaging group, Pediatric Neurology & Department of Neuroradiology, University Hospital, Tübingen, Germany; West China Hospital of Sichuan University, China

## Abstract

Subject motion has long since been known to be a major confound in functional MRI studies of the human brain. For resting-state functional MRI in particular, data corruption due to motion artefacts has been shown to be most relevant. However, despite 6 parameters (3 for translations and 3 for rotations) being required to fully describe the head's motion trajectory between timepoints, not all are routinely used to assess subject motion. Using structural (n = 964) as well as functional MRI (n = 200) data from public repositories, a series of experiments was performed to assess the impact of using a reduced parameter set (translation_only_ and rotation_only_) versus using the complete parameter set. It could be shown that the usage of 65 mm as an indicator of the average cortical distance is a valid approximation in adults, although care must be taken when comparing children and adults using the same measure. The effect of using slightly smaller or larger values is minimal. Further, both translation_only_ and rotation_only_ severely underestimate the full extent of subject motion; consequently, both translation_only_ and rotation_only_ discard substantially fewer datapoints when used for quality control purposes (“motion scrubbing”). Finally, both translation_only_ and rotation_only_ severely underperform in predicting the full extent of the signal changes and the overall variance explained by motion in functional MRI data. These results suggest that a comprehensive measure, taking into account all available parameters, should be used to characterize subject motion in fMRI.

## Introduction

Subject motion has long since been known to be a major confound in functional MRI studies of the human brain [1]. For resting-state functional MRI (rsfMRI) and functional connectivity analyses in particular, even minimal motion was recently found to be highly problematic [2]–[5]. Both prospective [6]–[8] and retrospective approaches [9], [10] to motion correction have been suggested, but the most commonly-used approach still is retrospective “motion correction” by using a rigid-body translation [11], [12]. However, even after such a procedure, motion still explains substantial variance in the data [1], [14], [15]. Motion correction (a.k.a. realignment) is usually performed using the first (or mean) image of a dataset as the reference, providing a measure of absolute motion over a functional run [16]. However, it was suggested that the scan-to-scan (relative) motion may be more relevant, as slow motion may be both easier to correct and less detrimental to data quality [17]. As the thus-detected extent of subject motion is commonly used to identify and remove bad datasets (“motion scrubbing” [4], [18], [19]), accurately describing motion is most important.

**Figure 1:**
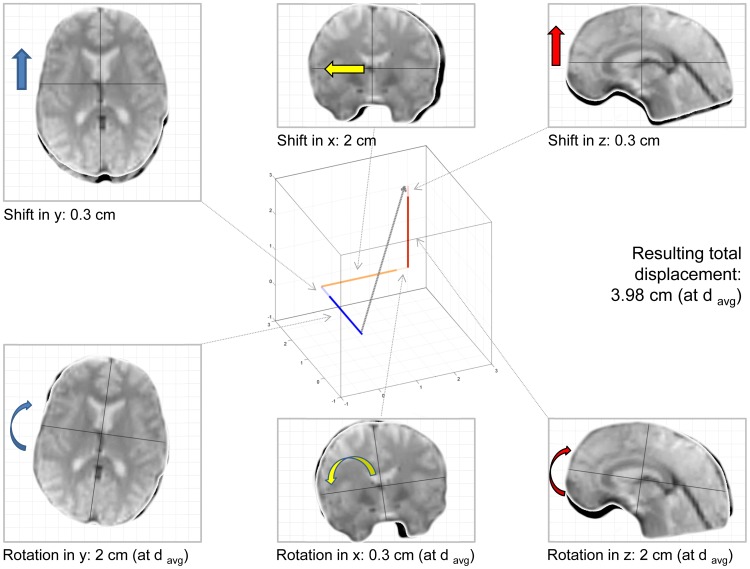
Illustration of the non-linear effects of combining translations (top row) and rotations (bottom row) into a single measure of total displacement (resulting gray arrow in the Cartesian coordinate system, middle). The values provided are only examples. Note that in this example, all displacements are additive, which is not always the case (see manuscript for more details). Note: d_avg_ is a measure of the average cortical distance, required to transform rotations to absolute distances.

During realignment, the aim is to find the combination of parameters that minimizes the difference between consecutive images, which may be defined using different cost functions [20]. The result of this rigid-body approach to motion correction is a set of 6 parameters. It is important to notice that these parameters are jointly optimized to achieve a final result; hence, only in their combination do they fully describe the motion trajectory detected by the realignment algorithm. However, assessing subject motion is only straightforward in the case of translations, which is described by 3 parameters (one for each dimension in space) and is provided in millimeters [mm]. In contrast to this, the assessment of subject rotation, (again described by 3 parameters but provided in degrees or radians), requires knowledge about the distance from the origin around which rotation was performed; only then are degrees/radians convertible to an absolute distance. It was suggested previously that the length of the vector resulting from these 6 transformations in space is an appropriate representation of subject motion ([15], [21], [22]; see Figure 1 for an illustration). This requires a definition of “at what distance” this motion is assessed, which may be the corner of the volume [22], set empirically (to 50 [4] or 65 mm [21]) or calculated individually [15]. This obstacle likely is responsible for many researchers qualifying “subject motion” by only inspecting absolute/relative translation, often applying a rule-of-thumb of “motion exceeding one voxel size” [12], [13], [23], [24]. As using only a subset of the complete realignment parameter set may systematically under- or overestimate motion and its effects, this study was aimed at addressing the following questions: I) what is a representative measure of cortical distance, and what is the effect of modifying it; II) to what extent does the isolated assessment of translation_only_ or rotation_only_ reflect true subject motion, as defined by total displacement; III) to what extent does the isolated assessment of translation_only_ or rotation_only_ affect data scrubbing procedures, i.e., when setting thresholds of acceptable subject motion; IV) to what extent does the isolated assessment of translation_only_ or rotation_only_ predict signal changes in the data; and V) to what extent does the isolated assessment of translation_only_ or rotation_only_ explain variance in the data, when compared with the complete assessment. This manuscript was not aimed to address these issues in such a way that solutions are presented, but rather to explore the presence, and potentially the magnitude, of the problem.

## Methods

To address the research questions posited above, both structural and functional MRI data was obtained from public data repositories. Structural MRI data was obtained from children (MRI dataset 1; The NIH study on normal brain development; n = 401 [25]) and adults (MRI dataset 2; IXI Study; n = 563 [26]); details of both datasets are described in Table 1 and are given in the Supplements S1 and S2. Functional MRI data (resting-state fMRI series) from adults was obtained by randomly picking 20 subjects each from 10 randomly selected participating sites' datasets from the fcon_1000 project (MRI dataset 3; n = 200 [27], [28]); details of this dataset are described in Table 2 and are given in the Supplement S3. All data processing steps and analyses were carried out in Matlab (version 8.2, The Mathworks, Natick, MA, USA), using custom scripts and functions as well as functionality provided within the SPM8 software package (Wellcome Trust Centre for Neuroimaging, University College London, UK). For all calculations, a 7^th^ order B-spline interpolation was used whenever possible [29] in order to avoid interpolation artefacts [30].

**Table 1:** Core characteristics of dataset 1 and 2 (structural MRI).

Dataset	Center	Subjects [n]	Voxel size [mm^3^]	Sex [M/F]	Ages [min-max]
Dataset 1 (NIH)	East	126	1.32±.61	61/65	4–17
	West	126	1.60±.48	62/64	4–18
	Midwest	149	1.43±.62	69/80	4–18
	Total Sample:	401	1.45±.59	192/209	10.6±3.48
Dataset 2 (IXI)	Guy's	313	1.025±.002	137/176	20–88
	IOP	70	1.025±0	24/46	20–81
	Hammersmith	180	1.025±0	87/93	20–86
	Total Sample:	563	1.025±.002	248/315	48.6±16.46

All data was acquired on scanners with a field strength of 1.5 Tesla, except for the Hammersmith Hospital data. Note that age is provided in years here, but was converted to “months at date of scan” for all calculations. Guy's, Guy's Hospital, London; IOP, Institute of Psychiatry, London; Hammersmith, Hammersmith Hospital, London. For more information on these datasets, see also Supplements S1 and S2.

**Table 2:** Core characteristics of dataset 3 (resting state functional MRI).

Dataset	Center	TR [msec]	Slices [n]	Volumes [n]	Sex [M/F/U]	Ages [min-max]
Dataset 3 (fcon_1000)	Atlanta, GA, USA	2000	20	205	6/14/1	22–54
	Baltimore, MD, USA	2500	47	123	7/13/0	20–40
	Bangor, UK	2000	34	265	20/0/0	19–38
	Beijing, China	2000	33	225	11/9/0	18–25
	Berlin, Germany	2300	34	195	12/8/0	23–44
	Cambridge, MA, USA	3000	47	119	3/17/0	18–24
	Cleveland, OH, USA	2800	31	127	8/12/0	24–57
	Dallas, TX, USA	2000	36	115	11/9/0	20–71
	ICBM, Montreal, Canada	2000	23	128	10/10/0	19–85
	Leiden, Netherlands	2180	38	215	16/4/0	20–27
					104/95/1	30.67±13.43

From each center, 20 subjects were selected at random (total n = 200); all data was acquired on scanners with a field strength of 3 Tesla. Note that age is provided in years here, but was converted to months at date of scan for all calculations. M, male; F, female; U, unknown. For more information on this dataset, see also Supplement S3.

### Experiment 1

The first experiment was aimed to address question I, what is a representative measure of cortical distance, and what is the effect of modifying it. For this experiment, MRI datasets 1 & 2 were used. The starting point here was the previously-suggested measure of average cortical distance [15], [21]. This indicator aims to provide a single number (distance from rotation origin) for which rotation can be converted to an absolute distance ([15], [21]; cf. Figure 1). In the motion fingerprint algorithm, it is calculated from each dataset individually [15], whereas in a commonly-used toolbox to assess motion effects in fMRI timeseries [31], this value is set empirically to 65 mm. While it is unclear as to whether this value is representative for a normal adult population, the situation is even less clear in the setting of developing brains, where substantial changes occur [25], [32]–[34]. To this effect, the combined structural MRI dataset of children and adults (total n = 964) was segmented into tissue classes using the unified segmentation approach implemented in SPM8 [35]. To rule out partial volume effects of different voxel sizes, the resulting native space gray matter tissue partitions were resliced to 1×1×1 mm isotropic resolution. Thereafter, all voxels on the outer cortical surface were identified and their absolute distance (in mm) to the image volume's point of origin was determined using a 3D extension of Pythagoras's theorem, as done before [15], yielding the Euclidian norm. These values were averaged, resulting in one value (average cortical distance, d_avg_) for each subject. These were then plotted according to age (in month at the time of data acquisition), and correlations with age were assessed as described below. Further, the effect of a difference in d_avg_ was investigated by modifying it in steps of.5 mm within a range of 50–80 mm as different values are used in the literature [4], [15], [31]. These values were then used to recalculate total displacement as well as scan-to-scan displacement (absolute and relative motion, respectively; see also below), for all subjects, using the results from d_avg_ = 65 mm as a reference.

### Experiment 2

The second experiment was aimed to address question II, to what extent does the isolated assessment of translation_only_ or rotation_only_ reflect true subject motion, as defined by total displacement. To this effect, MRI dataset 3 was used (resting state fMRI series, n = 200). Initially, a rigid-body realignment procedure was performed [11] as implemented in SPM8. Total displacement was calculated from the realignment parameters, as described above. Here, the spatial trajectory that minimizes the difference between the images and thus “corrects for” the individual subject's head motion is effectively recreated from the parameter set. From these 6 values, a vector in space is determined, the length of which (a.k.a. the Euclidian norm of the resulting 3-dimensional vector [22]) describes total displacement ([15], [21]; cf. Figure 1). The motion fingerprint algorithm [15] was used to assess absolute motion (total displacement, relative to the first volume) as well as relative motion (scan-to-scan displacement, relative to the previous volume) at the average cortical distance (d_avg_), here derived from the functional images. First, the original realignment parameters (6 parameters) were used; thereafter, values for either translation or rotation were set to 0, and calculations were repeated. This results in three displacement datasets (complete assessment [used as reference], translation_only_, and rotation_only_) and two resulting indicators (absolute and relative motion).

### Experiment 3

The third experiment was aimed to address question III, to what extent does the isolated assessment of translation_only_ or rotation_only_ affect data scrubbing procedures, when compared with the complete assessment dataset. This was explored by setting thresholds of acceptable subject motion, as done routinely in fMRI studies [4], [12], [13], [19], [24]. To this effect, cutoff values of.5/1/1.5/2/2.5/3 mm admissible motion were applied, again for both absolute and relative motion. Absolute and relative total displacement was calculated from the complete (used as reference) as well as the reduced (translation_only_, and rotation_only_) parameter sets. The number of datapoints exceeding these cutoff values was recorded and, for the reduced assessments, was related to the results from the complete assessment.

### Experiment 4

The fourth experiment was aimed to address question IV, to what extent does the isolated assessment of translation_only_ or rotation_only_ induce signal changes in the data. This was explored by again using the complete set of realignment parameters as well as the two reduced parameter sets (translation_only_ or rotation_only_) to recreate the subject's motion in a phantom timeseries. This timeseries is created by copying the first image in the timeseries *n* times and by then applying the inverted motion parameters from the *n* images to them (while simultaneously accounting for motion * B0 effects; [36]); this allows to assess the signal changes occurring as a function of motion. These signal changes are derived from 9 automatically-derived regions of interest in the brain [15], [37]; briefly, these are individually determined to be at the interface of brain and non-brain near the 8 corners of the image volume, as well as in the center of the brain. For this analysis, an average of the (absolute) timecourses from all 9 regions was used. The signal changes observable as a result of applying the reduced parameter sets were then again related to the changes resulting from applying the complete parameter set.

### Experiment 5

The fifth experiment was aimed to address question V, to what extent does the isolated assessment of translation_only_ or rotation_only_ explain variance in the data, when compared with the complete assessment parameter set. To this effect, different combinations of the reduced and complete assessment parameter sets were used as explanatory variables in a series of general linear model analyses (GLM [38]). The following parameter combinations were assessed: all realignment parameters from the complete assessment (rps_complete_), all realignment parameters from the translation_only_ assessment (rps_to_), and all realignment parameters from the rotation_only_ assessment (rps_ro_). For comparison purposes and following up on the results from experiment 4 (see below), the motion fingerprint (3 original and 3 traces, shifted back in time by one timepoint) from the complete assessment (mfp_complete_) as well as from both reduced assessments (mfp_to_ and mfp_ro_) was also included. These GLM-analyses were performed for every functional series in dataset 3. Thereafter, an omnibus F-test was used to assess the amount of variance explained by a given set of parameters [1], [15], [34]. It should be noted that this experiment is aimed to explore the relation of the variance explained by the complete and the reduced parameter sets; it is not aimed to exhaustively of formally compare the explanatory power of either approach. As a reference, the complete assessment set including two modifications (known as “Volterra expansions”) was used; to this effect, the original 6 realignment parameters were shifted back in time, and squared versions of each parameter were included, resulting in 24 parameters [1], [17]. This modified set was recently shown to explain the largest amount of variance in the data [15] and is therefore used as a reference (i.e., is set to 100%). Possible effects of loss of detection power [39], [40] and the fact that more parameters will by default explain more variance were not considered here.

### Statistics

Owing to considerations regarding non-linear interactions between parameters and non-normally distributed data, statistical comparisons were done using the non-parametrical Mann-Whitney-U-Test. Correlations were likewise assessed using Spearman's rank correlation. In order to avoid being vulnerable to the impact of unequal variances, heteroscedasticity was assessed using Henze-Zirkler's multivariate normality test, as implemented in the robust correlation toolbox [41]. In the presence of inhomogeneous variances, a skipped Spearman's correlation was calculated instead. Bootstrapped confidence intervals (CI) are given, providing further evidence that the correlation is not due to outliers alone. Significance was assumed at *p*≤.05, Bonferroni-corrected for multiple comparisons where appropriate.

## Results

### Experiment 1

**Figure 2:**
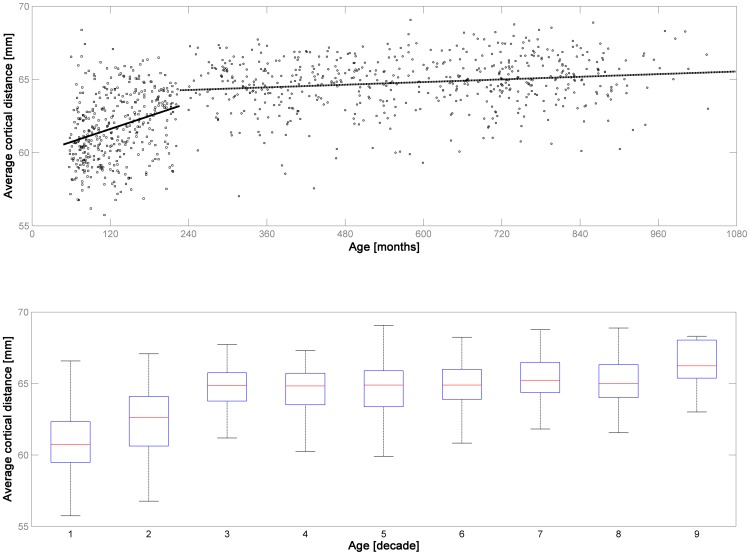
Illustration of the average cortical distance of all subjects in datasets 1 & 2 (structural MRI, n = 964). Note steep increase in childhood and adolescence (dataset 1, solid trendline in upper panel) and much more shallow increase in adulthood (dataset 2, dashed trendline in upper panel). Lower panel: illustration of the same results per decade of life. The difference between datasets 1 & 2 and of the first two decades with all other decades is significant (see manuscript for more details).

**Figure 3:**
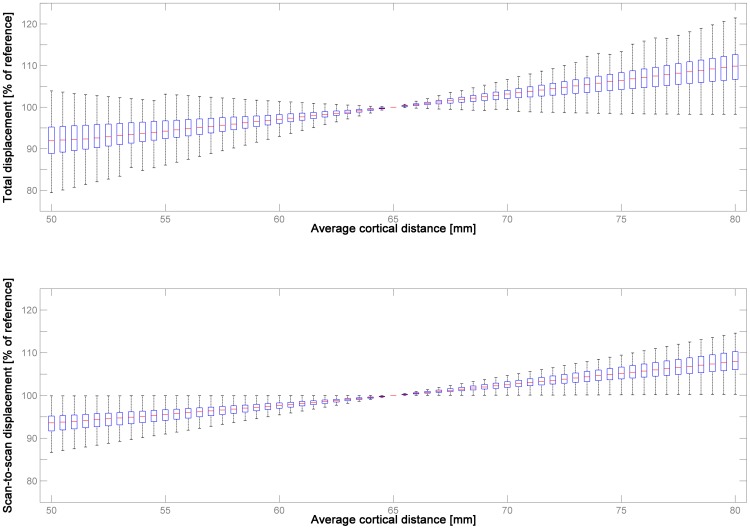
Illustration of the effect of varying average cortical distance on the resulting measure of absolute (total displacement, upper panel) and relative motion (scan-to-scan displacement, lower panel) in dataset 3 (n = 200), using 65 mm as a reference. Note systematic, but overall small effect, and substantial variability between subjects, underlining the inter-individual variation in ultimate motion trajectory composition.

When assessing the average cortical distance d_avg_ in the structural MR images in dataset 1 and 2, it is apparent that there is a clear developmental trend in childhood & adolescence (Figure 2), with d_avg_ increasing significantly with age (increase of.18 mm/year of age; skipped Spearman's r = .367 with CI = [.285–.452], *p*≤.001). Interestingly, there is a further increase in adulthood across the age range studied, but the slope is much less steep (increase of.015 mm/year of age; skipped Spearman's r = .1508 with CI = [.075–.232], *p*≤.001; Figure 2). When comparing the two datasets, there is a significant difference in d_avg_ in dataset 1 (children & adolescents, median = 61.58 mm) vs. dataset 2 (adults; median 64.95 mm; corrected *p*≤.001, Mann-Whitney-U-Test), as well as between the datasets from the first and second vs. all other decades (corrected *p*≤.05, Mann-Whitney-U-Test). The impact of systematically varying d_avg_ on both absolute and relative motion is illustrated in Figure 3.

### Experiment 2

**Figure 4:**
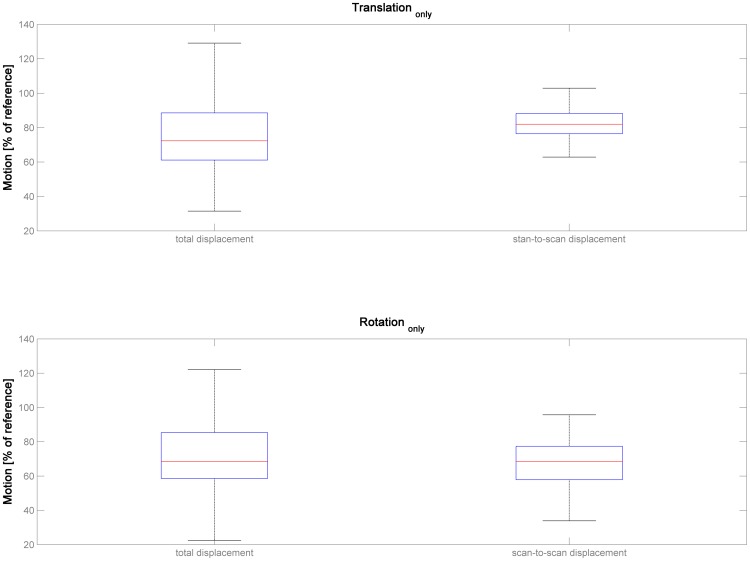
Illustration of estimated subject motion in dataset 3 (n = 200) for the two reduced parameter sets (translation_only_, top panels, and rotation_only_, bottom panels), for both indicators (total displacement, left panels, and scan-to-scan displacement, right panels). Note severe underestimation of total subject motion when compared with the full parameter set ( = 100%).

When comparing total displacement resulting from the complete assessment parameter set with the isolated assessment of translation_only_, it is apparent that the whole extent of subject motion is severely underestimated, for absolute (median = 72.3%, range 31.5–275.9) as well as for relative motion (median = 81.9%, range, 54.1–102.9; Figure 4). For both cases, this is significantly different from the complete parameter set (set to 100%; corrected *p*≤.001, Mann-Whitney-U-Test). A similar picture emerges when assessing total displacement resulting from rotation_only_ (absolute motion, median = 68.5%, range, 13.8–279.4; relative motion, median = 68.4%, range, 20.2–108.4). Again and for both cases, this is significantly different from the complete parameter set (set to 100%; corrected *p*≤.001, Mann-Whitney-U-Test).

### Experiment 3

When introducing a cutoff value to remove datapoints with unacceptable motion, both isolated assessments discard substantially less datapoints when compared with the results using the complete parameter set (Table 3). The effect initially becomes more pronounced at higher thresholds such that, on average, ∼31% (absolute motion) and ∼52% (relative motion) less voxels are discarded at a lower threshold (.5 mm), but ∼72% (absolute motion) and ∼77% (relative motion) less at a higher threshold (2 mm). Interestingly, the pattern reverses at the highest threshold (absolute motion, cutoff of 3 mm), such that the isolated assessment of both translation_only_ and rotation_only_ discard more datapoints then when using the complete parameter set.

**Table 3:** Summary of discarded datapoints per approach (from dataset 3, with total n = 34.340) and threshold, providing the relation to the assessment using the complete parameter set ( = 100%) as well as the corresponding absolute number of datapoints exceeding the threshold (n, values in parentheses).

		0.5 mm	1 mm	1.5 mm	2 mm	2.5 mm	3 mm
Complete assessment	absolute	100% (16.921)	100% (6.989)	100% (3.122)	100% (1.602)	100% (798)	100% (17)
	relative	100% (757)	100% (184)	100% (90)	100% (53)	100% (36)	100% (30)
Translation only	absolute	69.4% (11.749)	41.2% (2.881)	27.8% (870)	26.5% (425)	27.6% (221)	576.5% (98)
	relative	65.6% (497)	66.8% (123)	50% (45)	45.3% (24)	30.6% (11)	20% (6)
Rotation only	absolute	69.1% (11.693)	53.7% (3.760)	39.1% (1.221)	28.9% (463)	12.3% (98)	317.6% (54)
	relative	31.1% (236)	42.3% (78)	53.3% (48)	0% (0)	0% (0)	0% (0)

### Experiment 4

**Figure 5:**
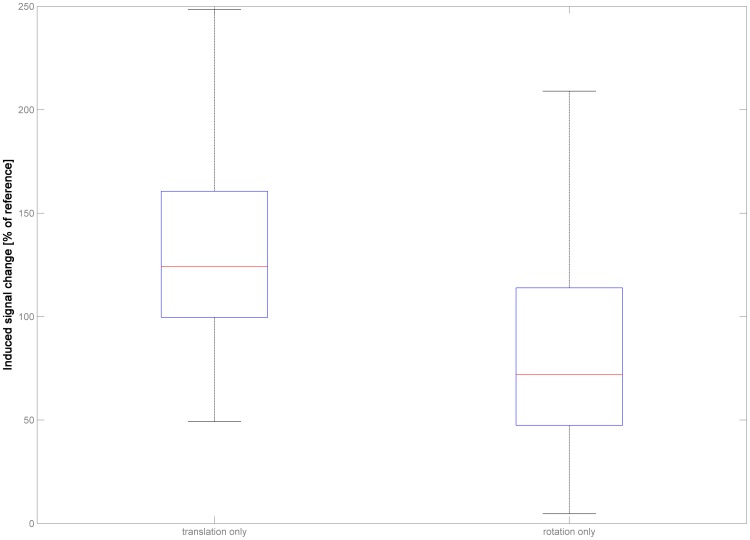
Illustration of the induced signal changes in dataset 3 (n = 200) for the two reduced parameter sets (translation_only_, left panel, and rotation_only_, right panel). Note severe deviation from expected observable signal changes when compared with the signal changes induced by the full parameter set ( = 100%).

When assessing the signal changes induced in the functional series in dataset 3 by re-applying the complete as well as the reduced parameter sets to a phantom timeseries, it is again apparent that there is no linear cause-effect relation (Figure 5). When assessing the signal changes induced by translation_only_, there is a notable increase in the observable signal changes over all subjects (median = 124.17%, range, 49.18–664.92). In contrast to this, the single changes induced by the rotation_only_ approach are substantially lower, albeit again with a wide spread (median = 71.98%, range, 4.68–627.86). For both cases, the difference is significant, as is the difference between the results from the two reduced parameter sets (all corrected *p*≤.001, Mann-Whitney-U-Test).

### Experiment 5

**Figure 6:**
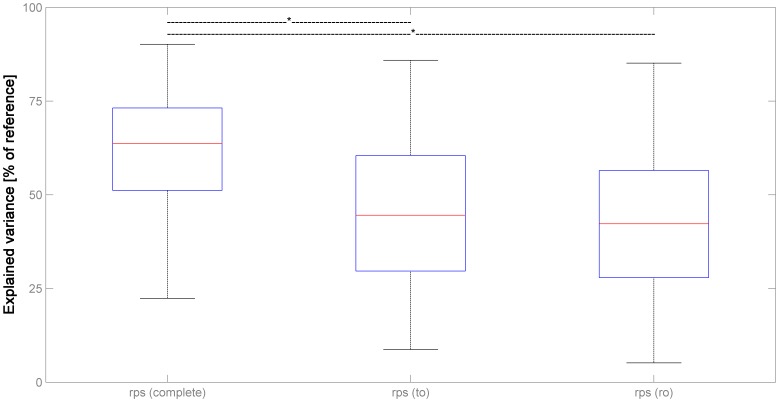
Illustration of the variance explained in dataset 3 (n = 200) by different parameter combinations: complete set of realignment parameters [rps (complete)], realignment parameters from translation_only_ [rps (to)] and rotation_only_ [rps (ro)]. Note increasingly severe underestimation of total motion-induced variance when compared with the full parameter set including Volterra expansion ( = 100%); see text for details.

When assessing the variance explained in the functional series by the complete as well as the reduced parameter sets, it is apparent that all complete and reduced parameter sets explain substantially and significantly less variance than the reference, Volterra-expanded complete parameter set (set to 100%; all corrected *p*≤.001, Mann-Whitney-U-Test; Figure 6). Further, the differences between the complete and the reduced realignment parameters sets also reach significance (corrected *p*≤.001, Mann-Whitney-U-Test). Interestingly, the difference between the complete and the reduced motion fingerprint parameter sets is much lower and does not reach significance.

## Discussion

This technical note was aimed at addressing the question of how well the effects of subject motion can be predicted when using a reduced parameter set (such as translation_only_).

The first experiment was aimed at assessing whether a representative value of the average cortical distance (d_avg_) could be derived from MRI data of both children and adults, to allow for the conversion of rotations into an absolute distance. As could be expected [25], [33], there is a clear developmental trend in children and adolescents, with a significant increase in d_avg_ (Figure 2). However, this finding is not as trivial as it may sound as brain size does not change substantially anymore [42] and linear scaling during spatial normalization does not correlate with age, in the age range studied [43]. Hence, global and local changes in tissue volume and shape as well as in gyrification could be to blame, with evidence for simultaneous progressive and regressive trends in either [32], [33], [44], [45]. The correlation of this distance parameter with age is actually also significant over the whole cohort in adults, but with a rather shallow slope and a low amount of explained variance. However, it is interesting to note that this correlation is likely brought about by an increase at the older end of the age spectrum, most prominently when comparing the 8^th^ and the 9^th^ decade (Figure 2), although it must be admitted that the individual numbers are small here. It is well known that local and global atrophy as well as changes in gyrification are also hallmarks or normal ageing [46]–[48]. One explanation for these two, seemingly contradictory observations could be that the predominating, opposing processes (increases in complexity in youth and cortical atrophy in ageing) lead to the same observable phenomena due to their impact on cortical morphology. However, it was felt that a further exploration of the underlying mechanisms was beyond the scope of this manuscript; hence, no further analyses were carried out.

When assessing the influence of modifying d_avg_, Figure 3 illustrates that the effect is, as expected, systematic, but surprisingly small. For example, when using d_avg_ = 60 mm instead of 65 mm, median absolute motion is 97.07% of the original, over all subjects; similarly, when using d_avg_ = 70 mm, it is 103.15%. These differences are slightly lower (97.69% and 102.50%, respectively), and less variable, for relative motion. Among the adults included here, 98.8% were within the range of 60–70 mm, and still 73% of the children and adolescents. While these median differences are small, there is a certain variability, which becomes wider when moving further away from the suggested value of 65 mm. This increase in variability can only be due to rotations and underlines that the relation between translations and rotations is highly individual to each subject, as seen before [23]. Hence, a systematic bias may indeed result when comparing subjects with a systematically differing d_avg_, such as children vs. adults, as motion will either be slightly underestimated in children or slightly overestimated in adults. On the other hand, these results also suggest that the magnitude of the imprecision induced by using a single, empirically derived value of 65 mm [21] will be rather small, even when assessing a wide range of normal (adult or pediatric) subjects (cf. Figure 2). Using a single indicator has the advantage of making results more comparable between subjects and populations, and it precludes being vulnerable to miscalculations from the actual data [15], for example when the available fMRI data only covers part of the brain, as in high-resolution studies [49], [50]. Consequently, this value can be considered to be both useful and representative.

The second experiment was aimed to address the relation of subject motion when using the complete parameter set versus when assessing translation or rotation in isolation. The results demonstrate that the true extent of subject motion is underestimated by a median of ∼20–30% when looking at translation_only_ or rotation_only_ (Figure 4). This effect can be observed for both absolute and relative motion. Interestingly, motion is not exclusively underestimated in both reduced parameter sets: while the median is substantially lower, there are also several datapoints exceeding 100% in both analyses. This underlines that the relation of both sets of parameters is not simply additive: accounting for rotation may mean that the motion estimated from translation_only_ is actually reduced, and vice versa. In fact, when assessing the corresponding dimensions (shifts & rotations in x, y, and z) in the whole functional MRI dataset, every single subject shows a substantial number of datapoints with opposite signs between these two parameters. Specifically, in 16.044 [x], 15.641 [y], and 15.385 [z], respectively, of the 34.340 datapoints [per dimension], a shift with a positive sign was accompanied by a rotation with a negative sign, or vice versa. It is therefore important to notice that this complex interrelation precludes an extrapolation of total motion from either factor (as in “total motion≈translation * x”, with x representing a fixed factor). This further argues for a combined assessment.

The effect of using a reduced parameter set for quality control purposes was addressed in experiment 3. As can be seen from Table 3, substantially fewer datapoints are discarded when applying a cutoff value in the isolated analyses of translation_only_ or rotation_only_ in almost all scenarios, when compared with using the full parameter set. However, the effect may actually reverse, as can be seen at higher thresholds (Table 3, right-most column). This further underlines the non-linear nature of the interaction of the two reduced parameter sets and again suggests that using translation_only_ or rotation_only_ to assess data quality in functional MRI studies is of only limited applicability, and may be misleading.

In order to assess the effects of motion on the actual fMRI data, the signal change induced by motion can be estimated by reproducing motion in phantom timeseries [15]. This was investigated here in experiment 4, again using the complete parameter set as the reference for the two reduced sets. It is interesting to notice that translation_only_ actually leads to stronger signal changes in the data, while rotation_only_ induces significantly weaker signal changes, when compared with signal changes induced by the complete parameter set (Figure 5). This again points toward the non-linear interrelation of both reduced parameter sets: while they may in some cases be additive, they may also be subtractive (which, as laid out above, is the case in ∼45% of datapoints). It should be noted that the signal changes resulting from the interaction of the head with the static magnetic field (motion * B0 interaction [36], [51]) are automatically computed in our motion fingerprint approach. The impact of using a reduced parameter set on this procedure has not been evaluated here. Irrespective of the exact contribution of the different sources, though, these results suggest that the extent of either parameter in isolation is not reliably predictive of the to-be-expected signal change in functional MRI data.

When assessing the amount of variance explained by the different parameter sets in experiment 5, the lower variance explained by the 6 realignment parameters when compared with the Volterra-expanded version confirms previous results [1], [15], [17]. However, the reduced parameter sets (translation_only_ and rotation_only_) explain significantly less variance again (Figure 6). The difference between the original motion fingerprint approach and the complete realignment parameter set is not significant, again in line with previous results [15]. It is interesting to note, though, that the variance explained by the motion fingerprint does not change as much when using the reduced parameter sets. This is likely due to the fact that, although the reduced parameter sets underestimate subject motion per se (cf. Figure 4), they may both over- and underestimate the resulting signal changes (cf. Figure 5). These discrepancies seem to cancel out to the effect that, overall, the variance explained in the reduced analyses does not differ significantly from the original analysis. On a side note and again confirming previous results [15], the variance explained by a complete motion fingerprint (9 traces) including shifted versions was not significantly lower (median = 93.93%, data not shown) than the variance explained by the reference dataset (Volterra-expanded motion parameters; [1], [17]). Taken together, these results suggest that either reduced parameter set in isolation does not reliably predict the variance explained by subject motion in functional MRI data.

## Limitations

For this study, several large datasets were used, providing a robust assessment of the resulting metrics, but as always, there are limitations. For one, segmentation of pediatric imaging data should ideally not be performed using adult reference data [34], [52]; in order to allow comparability of results over both (adult & pediatric) datasets in experiment 1, the potentially resulting inaccuracies were considered to be secondary. Further, the isolation of the realignment parameters for translation_only_ and rotation_only_ was done post-hoc, and it could be argued that the realignment algorithm should be constrained *a priori* to only perform motion correction using either in isolation. Alternatively, a completely synthetic motion effects simulator approach could be used [51]. On the other hand, the current manuscript investigates a realistic scenario, and being closer to a real-life setting was ultimately judged to be more important. It should also be noted that only one approach to motion correction (the one implemented in SPM8) was used here, while several other implementations are available, e.g. [20], [53]–[55]; however, this manuscript was aimed at highlighting the different shortcomings of using a reduced parameter set to assess subject motion, and the main results are likely independent of the technical implementation of the algorithm, and thus generalizable. Also, no fMRI data acquired in special settings (such as high-motion datasets from patients [17], tasks involving overt speech [56], or data from children [15]) was investigated here. In fact, no dataset using task-based functional MRI was investigated here, which disallows assessing the impact of using different strategies on the resulting statistical maps; however, this was done before [1], [15], [17], [19], [54]; besides, using resting-state fMRI data has the added benefit of avoiding the potential interaction of task-induced activation with motion correction [23].

## Conclusions

Subject motion is “corrected for” by using a rigid body procedure, which is described in full only by all 6 translation *and* rotation parameters. The results presented here suggest that these two reduced parameter sets (translation_only_ and rotation_only_) can be combined in a meaningful way, using 65 mm as a representative and useful approximation of the average cortical distance. The thus-resulting total displacement cannot be reliably approximated using either reduced parameter set. Therefore, motion censoring procedures relying on a reduced parameter set do not seem appropriate, and both signal changes induced and variance explained by subject motion are severely underestimated. Consequently, a comprehensive measure, taking into account all parameters, should be used to characterize subject motion in fMRI.

## Supporting Information

Supplement S1 **Includes the detailed listing of all subject IDs from dataset 1 that were used in this study.**
(DOCX)

Supplement S2 **Includes the detailed listing of all subject IDs from dataset 2 that were used in this study.**
(DOCX)

Supplement S3 **Includes the detailed listing of all subject IDs from dataset 3 that were used in this study.**
(DOCX)
