# Supplementary material for: Isolated Assessment of Translation or Rotation Severely Underestimates the Effects of Subject Motion in fMRI Data
Source: PLoS One. 2014 Oct 21;9(10):e106498. doi: 10.1371/journal.pone.0106498 (PMC4204812; doi:10.1371/journal.pone.0106498)
Supplement: Supplement S1 — Includes the detailed listing of all subject IDs from dataset 1 that were used in this study. (DOCX) [file pone.0106498.s001.docx]

**Isolated assessment of translation or rotation severely underestimates the effects of subject motion in fMRI data**

Marko Wilke ^1, 2^

^1^ Department of Pediatric Neurology & Developmental Medicine, Children’s Hospital

^2^ Experimental Pediatric Neuroimaging group, Pediatric Neurology & Department of Neuroradiology, University Hospital, Tübingen, Germany

**Supplement 1: detailed listing of dataset 1**

From the *NIH Study of normal brain development* (NIH, Bethesda, MD, USA), available at <http://pediatricmri.nih.gov/nihpd/info/index.html>, subjects with the following IDs were used: 1001, 1002, 1003, 1004, 1005, 1007, 1008, 1009, 1010, 1011, 1012, 1013, 1014, 1015, 1016, 1017, 1018, 1019, 1020, 1021, 1023, 1024, 1025, 1026, 1027, 1028, 1029, 1030, 1031, 1032, 1033, 1034, 1035, 1036, 1037, 1038, 1039, 1040, 1041, 1042, 1043, 1044, 1045, 1046, 1047, 1051, 1052, 1053, 1054, 1055, 1056, 1057, 1058, 1061, 1062, 1063, 1064, 1065, 1066, 1067, 1068, 1069, 1070, 1071, 1072, 1073, 1074, 1075, 1076, 1077, 1078, 1079, 1080, 1081, 1082, 1083, 1084, 1086, 1087, 1088, 1089, 1090, 1091, 1092, 1093, 1094, 1095, 1096, 1097, 1098, 1099, 1100, 1102, 1103, 1104, 1105, 1106, 1107, 1108, 1109, 1110, 1111, 1112, 1113, 1114, 1115, 1116, 1118, 1119, 1121, 1122, 1123, 1124, 1125, 1126, 1127, 1128, 1129, 1130, 1131, 1132, 1133, 1134, 1136, 1138, 1139, 1140, 1141, 1142, 1143, 1144, 1145, 1146, 1147, 1148, 1149, 1150, 1151, 1153, 1154, 1155, 1157, 1158, 1159, 1160, 1161, 1162, 1163, 1164, 1165, 1166, 1167, 1168, 1169, 1170, 1171, 1172, 1173, 1174, 1175, 1177, 1178, 1179, 1180, 1181, 1182, 1183, 1184, 1185, 1186, 1187, 1188, 1189, 1190, 1191, 1192, 1193, 1194, 1195, 1196, 1197, 1199, 1200, 1201, 1202, 1203, 1204, 1205, 1206, 1207, 1208, 1209, 1210, 1211, 1213, 1214, 1215, 1216, 1217, 1218, 1219, 1220, 1221, 1222, 1223, 1224, 1225, 1226, 1227, 1228, 1229, 1230, 1231, 1232, 1233, 1234, 1235, 1236, 1237, 1238, 1239, 1240, 1241, 1242, 1243, 1244, 1245, 1246, 1247, 1248, 1249, 1250, 1251, 1252, 1253, 1254, 1255, 1257, 1258, 1259, 1260, 1262, 1263, 1264, 1266, 1267, 1268, 1269, 1270, 1271, 1272, 1273, 1274, 1275, 1276, 1277, 1278, 1279, 1280, 1281, 1282, 1283, 1284, 1285, 1286, 1287, 1288, 1289, 1290, 1291, 1292, 1294, 1297, 1298, 1299, 1300, 1301, 1302, 1303, 1304, 1305, 1306, 1307, 1308, 1309, 1310, 1311, 1312, 1313, 1314, 1315, 1316, 1317, 1318, 1319, 1320, 1321, 1322, 1323, 1324, 1325, 1326, 1328, 1329, 1330, 1331, 1332, 1333, 1334, 1335, 1336, 1337, 1338, 1339, 1340, 1341, 1342, 1343, 1344, 1346, 1348, 1349, 1350, 1351, 1352, 1353, 1354, 1355, 1356, 1357, 1358, 1359, 1360, 1361, 1362, 1363, 1364, 1365, 1366, 1367, 1368, 1369, 1370, 1371, 1372, 1373, 1374, 1375, 1376, 1377, 1378, 1379, 1380, 1381, 1382, 1383, 1384, 1385, 1386, 1387, 1388, 1389, 1390, 1391, 1392, 1393, 1395, 1396, 1397, 1398, 1399, 1400, 1401, 1402, 1403, 1404, 1405, 1406, 1407, 1408, 1410, 1411, 1413, 1414, 1415, 1416, 1417, 1418, 1419, 1420, 1422, 1423, 1424, 1425, 1426, 1427, 1428, 1429, 1430, 1431, 1432.

The following disclaimer applies: this manuscript reflects the views of the author and may not reflect the opinions or views of the Brain Development Cooperative Group Investigators or the NIH. The contract numbers for the NIH MRI study of normal brain development were N01-HD02-3343, N01-MH9-0002, and N01-NS-9-2314, 2315, 2316, 2317, 2319 and 2320. A listing of the participating sites and a complete listing of the study investigators can be found at the website of the data coordinating center at [www.bic.mni.mcgill.ca/nihpd/info/participating_centers.html](http://www.bic.mni.mcgill.ca/nihpd/info/participating_centers.html).
