# Supplementary material for: Isolated Assessment of Translation or Rotation Severely Underestimates the Effects of Subject Motion in fMRI Data
Source: PLoS One. 2014 Oct 21;9(10):e106498. doi: 10.1371/journal.pone.0106498 (PMC4204812; doi:10.1371/journal.pone.0106498)
Supplement: Supplement S2 — Includes the detailed listing of all subject IDs from dataset 2 that were used in this study. (DOCX) [file pone.0106498.s002.docx]

**Isolated assessment of translation or rotation severely underestimates the effects of subject motion in fMRI data**

Marko Wilke ^1, 2^

^1^ Department of Pediatric Neurology & Developmental Medicine, Children’s Hospital

^2^ Experimental Pediatric Neuroimaging group, Pediatric Neurology & Department of Neuroradiology, University Hospital, Tübingen, Germany

**Supplement 2: detailed listing of dataset 2**

From the *IXI-Dataset, Biomedical Image Analysis Group* (Imperial College, London, UK), available at <http://biomedic.doc.ic.ac.uk/brain-development/index.php?n=Main.Datasets>, subjects with the following IDs were used: 002, 012, 013, 014, 015, 016, 017, 019, 020, 021, 022, 023, 024, 025, 026, 027, 028, 029, 030, 031, 033, 034, 035, 036, 037, 038, 039, 040, 041, 042, 043, 044, 045, 046, 048, 049, 050, 051, 052, 053, 054, 055, 056, 057, 058, 059, 060, 061, 062, 063, 064, 065, 066, 067, 068, 069, 070, 071, 072, 073, 074, 075, 076, 077, 078, 079, 080, 083, 084, 085, 086, 087, 089, 090, 091, 092, 093, 094, 095, 096, 097, 098, 099, 100, 101, 102, 103, 104, 105, 106, 107, 108, 109, 110, 111, 112, 113, 114, 115, 116, 118, 119, 120, 121, 122, 123, 126, 127, 128, 129, 130, 131, 132, 134, 135, 136, 137, 138, 139, 140, 141, 142, 143, 144, 145, 146, 148, 150, 151, 153, 154, 156, 157, 158, 159, 160, 161, 162, 163, 164, 165, 166, 167, 168, 169, 170, 172, 173, 174, 175, 176, 177, 178, 179, 180, 181, 182, 183, 184, 185, 186, 188, 189, 191, 192, 193, 194, 195, 196, 197, 198, 199, 200, 201, 202, 204, 205, 206, 207, 208, 209, 210, 211, 212, 213, 214, 216, 217, 218, 219, 221, 222, 223, 224, 225, 226, 227, 229, 230, 231, 232, 233, 234, 236, 237, 238, 239, 240, 241, 242, 244, 246, 247, 248, 249, 250, 251, 252, 253, 254, 255, 256, 257, 258, 259, 260, 261, 262, 263, 264, 265, 266, 267, 268, 269, 270, 274, 275, 276, 277, 278, 279, 280, 282, 284, 285, 286, 287, 288, 289, 290, 291, 292, 293, 294, 295, 296, 297, 298, 299, 300, 302, 303, 304, 305, 306, 307, 308, 309, 310, 311, 312, 313, 314, 315, 316, 317, 318, 319, 320, 321, 322, 324, 325, 326, 327, 328, 329, 330, 331, 332, 334, 335, 336, 337, 342, 344, 348, 350, 351, 353, 354, 356, 357, 358, 359, 360, 361, 362, 363, 364, 365, 367, 368, 369, 370, 371, 372, 373, 375, 376, 377, 378, 379, 380, 381, 382, 383, 384, 385, 386, 387, 388, 389, 390, 391, 392, 393, 394, 395, 396, 397, 398, 399, 400, 401, 402, 403, 404, 405, 406, 407, 408, 409, 410, 411, 412, 413, 414, 415, 416, 417, 418, 419, 420, 422, 423, 424, 425, 426, 427, 428, 429, 430, 431, 432, 433, 434, 435, 436, 437, 438, 439, 440, 441, 442, 443, 444, 445, 446, 447, 448, 449, 450, 451, 452, 453, 454, 455, 456, 457, 458, 459, 460, 461, 462, 463, 464, 465, 467, 468, 469, 470, 473, 474, 475, 476, 477, 478, 479, 480, 481, 482, 483, 484, 485, 486, 487, 488, 489, 490, 491, 492, 493, 494, 495, 496, 497, 498, 499, 500, 503, 504, 505, 506, 507, 508, 510, 511, 512, 515, 516, 517, 518, 519, 521, 522, 523, 524, 525, 526, 527, 528, 531, 532, 533, 534, 535, 536, 537, 538, 539, 541, 542, 543, 544, 546, 547, 548, 549, 550, 551, 552, 553, 554, 555, 556, 558, 559, 560, 561, 562, 563, 565, 566, 567, 568, 569, 571, 572, 573, 574, 575, 576, 577, 578, 579, 582, 584, 585, 586, 587, 588, 591, 592, 593, 594, 595, 596, 597, 598, 599, 600, 601, 603, 605, 606, 607, 608, 609, 610, 611, 612, 613, 614, 616, 617, 618, 619, 621, 622, 625, 626, 627, 629, 630, 631, 632, 633, 634, 635, 636, 639, 640, 641, 642, 644, 646, 648, 651, 652, 653, 662.
