# Supplementary material for: Isolated Assessment of Translation or Rotation Severely Underestimates the Effects of Subject Motion in fMRI Data
Source: PLoS One. 2014 Oct 21;9(10):e106498. doi: 10.1371/journal.pone.0106498 (PMC4204812; doi:10.1371/journal.pone.0106498)
Supplement: Supplement S3 — Includes the detailed listing of all subject IDs from dataset 3 that were used in this study. (DOCX) [file pone.0106498.s003.docx]

**Isolated assessment of translation or rotation severely underestimates the effects of subject motion in fMRI data**

Marko Wilke ^1, 2^

^1^ Department of Pediatric Neurology & Developmental Medicine, Children’s Hospital

^2^ Experimental Pediatric Neuroimaging group, Pediatric Neurology & Department of Neuroradiology, University Hospital, Tübingen, Germany

**Supplement 3: detailed listing of dataset 3**

From *The 1000 Functional Connectomes Projec*t), available at <http://www.nitrc.org/ir/app/action/ProjectDownloadAction/project/fcon_1000>, subjects with the following IDs were used: from Atlanta: 00354, 00368, 07145, 15817, 18219, 18702, 24972, 32093, 52783, 53122, 58250, 59806, 60499, 61442, 71337, 72096, 75153, 76280, 86323, 91049; from Bangor: 00031, 01903, 03557, 04097, 14388, 27519, 36736, 46870, 48632, 61418, 61908, 63767, 66585, 68050, 73082, 77520, 81464, 82625, 87568, 91556; from Beijing: 00440, 08455, 17603, 20765, 26713, 29590, 31058, 36580, 43290, 48501, 50972, 55856, 62438, 66889, 69696, 74386, 80569, 85030, 91952, 98617; from Baltimore: 17017, 23927, 29158, 30072, 31837, 37548, 52358, 54257, 54329, 73823, 76160, 80221, 81887, 85922, 86414, 90658, 90893, 91622, 94042, 96234; from Berlin: 06204, 06716, 12855, 18913, 27536, 27711, 27797, 33248, 38279, 40143, 47791, 49134, 54976, 67166, 75506, 77281, 86111, 91116, 91966, 97162; from Cambridge: 00156, 02591, 06037, 08588, 13216, 17584, 24670, 29800, 42146, 49998, 53615, 58682, 61209, 65682, 72068, 77989, 83683, 89435, 93609, 99330; from Cleveland: 02480, 07835, 13495, 18011, 19005, 20003, 22935, 28596, 34189, 46075, 47482, 50092, 61868, 64706, 67936, 75398, 80263, 85091, 92232, 97844; from Dallas: 04288, 05892, 16493, 25085, 32183, 32272, 40897, 51824, 57450, 58347, 58803, 71043, 77150, 79426, 80418, 81423, 83998, 88725, 89418, 92054; from Montreal: 00448, 05208, 10582, 16607, 26183, 28808, 32549, 35262, 40217, 44077, 49215, 55114, 59914, 68850, 77431, 85442, 87217, 93262, 94945, 98802; and from Leiden: 01553, 01787, 08518, 09796, 10481, 12255, 13537, 18456, 19281, 28473, 36743, 38454, 39335, 40907, 52853, 52922, 56299, 57187, 58194, 99856.
